# Effectiveness of epidural anaesthesia induced by different dosages of ropivacaine combined with lidocaine in geriatric orthopaedic surgery

**DOI:** 10.1308/rcsann.2025.0055

**Published:** 2025-07-29

**Authors:** J Li, Q Yao, J Shi, P Jiang

**Affiliations:** ^1^ Affiliated Hospital of Jiangsu University, China; ^2^ Nanjing University of Chinese Medicine, China

**Keywords:** Epidural anaesthesia, Ropivacaine, Lidocaine, Geriatric orthopaedic surgery, Heart rate, Mean arterial pressure, Central venous pressure

## Abstract

**Objective:**

This paper unravelled the effectiveness of epidural anaesthesia induced by different dosages of ropivacaine combined with lidocaine in geriatric orthopaedic surgery.

**Methods:**

Elderly orthopaedic surgical patients who underwent epidural anaesthesia were allocated into three groups: Group I (0.375% ropivacaine+1% lidocaine), Group II (0.50% ropivacaine+1% lidocaine) and Group III (0.75% ropivacaine+1% lidocaine), to compare the sensory and motor blockade effects, the occurrence of cognitive function and cognitive dysfunction, and the occurrence of adverse reactions.

**Results:**

The onset time of sensory blockade and the appearance time of the highest plane were significantly earlier and the maintenance time of the highest plane was significantly longer, the onset time of motor blockade was notably earlier, the maintenance time of the highest plane was markedly longer, and the modified Bromage score was distinctly higher in Group II and Group III in comparison with Group I (*p*<0.05). The postoperative 12- and 24-h Mini-Mental State Examination scores were higher and the incidence of postoperative 12h-cognitive impairment was lower in Group III than in Group I and Group II (*p*<0.05). There was no statistically significant difference in the incidence of adverse reactions in patients among the three groups (*p*>0.05).

**Conclusions:**

Epidural anaesthesia combining 0.75% ropivacaine with 1% lidocaine in elderly orthopaedic surgical patients has a rapid onset of action, a long duration of anaesthesia maintenance, a low overall impact on cognitive function, and good medication safety.

## Introduction

With the increase in the global elderly population, the demand for surgery and the total expenditure on medical insurance is also expected to increase.^1^ The aging trend in China indicates that the aging level of the population is likely to intensify, and influence the balance of future medical insurance funds.^2^ There is a growing awareness that geriatric patients will have multiple chronic conditions, which restricts their functional capacity and recovery.^3^ Perioperative complications and mortality remain higher in geriatric patients than in adult patients despite advances in anaesthetic and surgical techniques.^4,5^ The number of elderly orthopaedic patients is growing steadily and all measures should be taken by anaesthesiologists to allow early and effective rehabilitation, a concept that is now broadly recognised to enhance the success of orthopaedic surgery.^6^ To provide optimal care for elderly surgical patients, a comprehensive evaluation of the health status of a patient must be performed to identify and resolve deficits.

Elderly patients represent an increasing proportion of anaesthesia practice, especially in orthopaedic surgery.^7^ Methods for regional anaesthesia consist of epidural anaesthesia and spinal anaesthesia, as well as combined spinal epidural anaesthesia.^8^ Epidural anaesthesia is characterised by reduced interference on the respiratory and circulatory system, diminished physiological impact, quick postoperative recovery, as well as fewer complications.^9^ Epidural anaesthesia is associated with reduced blood loss in orthopaedic surgery.^10^ Some data reveal that intraoperative epidural anaesthesia improves the quality of life and reduces neuroinflammatory responses after oesophagectomy in elderly patients compared with patients receiving only general anaesthesia.^11^ Due to the haemodynamic effects of epidural anaesthesia on the visceral organs that are less tolerated by the elderly, more major complications may occur with the use of epidural anaesthesia. Therefore, more studies of epidural interventions are needed to determine the impact of epidural anaesthesia on individual procedures.^10^

Ropivacaine is known as a novel long-acting amide local anaesthetic, which has been applied for subcutaneous infiltration and postoperative analgesia, along with intrathecal, epidural and peripheral nerve block surgery since its introduction in 1996.^12^ Although adverse reactions are rare when it is administered properly, its overuse may have adverse effects on the cardiovascular and central nervous systems, including drowsiness, arrhythmias, loss of consciousness and cardiac arrest.^13^ Therefore, appropriate drug dose is the key to limiting the potential adverse reactions of ropivacaine in elderly patients. Lidocaine, as an amide local anaesthetic, was initially utilised intravenously as an antiarrhythmic drug. To some extent, it has been suggested that intravenous lidocaine has analgesic effects and may be beneficial in perioperative settings.^14^ Lidocaine has long been applied for the treatment of diverse pain disorders, and it has anti-inflammatory properties in addition to pain control.^15^ Evidence has shown that caudal anaesthesia of ropivacaine plus lidocaine is a safe and effective induction of anaesthesia during haemorrhoidectomy.^16^ In this paper, we aimed to unravel the effectiveness of epidural anaesthesia induced by different dosages of ropivacaine combined with lidocaine in geriatric orthopaedic surgery.

## Methods

### Ethical approval

Written informed consent was acquired from the patients and their families and the research was approved by the ethics committee of our hospital.

### Experimental subjects

Geriatric orthopaedic patients attending our hospital from January 2020 to December 2022 were selected. Inclusion criteria were (1) patients ≥60 years old; (2) patients with no preoperative speech or cognitive impairment; (3) those who cooperated voluntarily with the study; and (4) those with complete clinical data. Exclusion criteria were (1) patients with contraindications to surgery; (2) those with a history of drug allergy; (3) those with abnormalities in hepatic and renal function; (4) those with psychiatric disorders; (5) those who had used any analgesic within 24h before the study; (6) those with recent use of adrenoceptor agonists, antagonists and opioids; and (7) those with a history of cardiac disease. After rigorous screening by the inclusion and exclusion criteria, 153 cases of patients were finally entered into this study. These 153 patients were assigned by computer-generated randomised sequence to three groups (1:1:1): Group I (0.375% ropivacaine+1% lidocaine), Group II (0.50% ropivacaine+1% lidocaine) and Group III (0.75% ropivacaine+1% lidocaine), with 51 cases in each group.

### Experimental methods

The patients were fasted for 8h and dehydrated for 2h before the operation. After entering the operating room, intravenous access was gained, 300–500ml of sodium lactate ringer’s solution was given to the patient for input and vital signs were monitored. An epidural anaesthesia catheter was placed and, after the epidural injection of 2ml of 1% lidocaine, the patient’s condition was observed and local anaesthesia was administered to determine that there were no discomfort reactions. Group I (0.375% ropivacaine+1% lidocaine) patients were given a 0.375% ropivacaine (dose 7.5mg) and 1% lidocaine (2ml) mixture; the Group II (0.50% ropivacaine+1% lidocaine) patients were given 0.50% ropivacaine (dose 10mg) and 1% lidocaine (2ml) mixture; the Group III (0.75% ropivacaine+1% lidocaine) patients were given 0.75% ropivacaine (dose 15mg) and 1% lidocaine (2ml) mixture. Vital signs such as heart rate and blood pressure were monitored during anaesthesia. If the patient had a systolic blood pressure below 90mmHg during anaesthesia, an intravenous injection of ephedrine (5–10mg) was required. If the patient’s heartrate was less than 60 beats/min, an intravenous injection of 0.5mg atropine was required to ensure the stability of the patient’s vital signs during anaesthesia.

### Observation indicators

1.  Sensory blockade effect (onset time of sensory blockade, appearance time of the highest plane and maintenance time of the highest plane).
2.  Motor blockade effect (onset time of motor blockade, maintenance time of motor blockade and modified Bromage score).
3.  Heart rate and mean arterial pressure (MAP) at different timepoints (before anaesthesia, 30min after anaesthesia and 2h after surgery).
4.  Cognitive function and occurrence of cognitive impairment. The Mini-Mental State Examination (MMSE, 0–30 points)^17^ was used for cognitive function evaluation.
5.  Adverse reactions (bradycardia, hypotension, respiratory depression, nausea and vomiting).

### Evaluation criteria for the modified Bromage score

Modified Bromage scores are rated points: 0 points: the patient had no sensory and motor blockade; 1 point: the patient could not lift their legs; 2 points: the patient could not bend their knees; 3 points: the patient could not bend the ankle joint.

### Statistical analysis

The data were analysed using SPSS version 24.0 statistical software. Categorical data were expressed as *n* (%), which were analysed by χ2-test or Fisher’s exact test. Measurement data were represented by mean±standard deviation, and compared by analysis of variance (ANOVA); *p*<0.05 indicated a statistically significant difference.

## Results

### General information

The differences in age, sex ratio, body mass index and disease type among the Group I (0.375% ropivacaine+1% lidocaine), Group II (0.50% ropivacaine+1% lidocaine) and Group III (0.75% ropivacaine+1% lidocaine) were not statistically significant (*p*>0.05) and were comparable (Table 1).

**Table 1:** Comparison of general data in Groups I–III

Indicator	Group I (*n*=51)	Group II (*n*=51)	Group III (*n*=51)	*p* value
Age (years)	66.27±4.96	67.65±4.85	67.63±5.19	0.286
Sex				0.711
Male	25	28	29	
Female	26	23	22	
Weight (kg)	58.10±7.36	57.28±7.14	58.39±7.83	0.737
Disease type				0.947
Knee osteoarthritis	9	10	9	
Femoral neck fracture	25	26	23	
Intertrochanteric fracture	17	15	19	

### Sensory blockade effect

**Figure 1:**
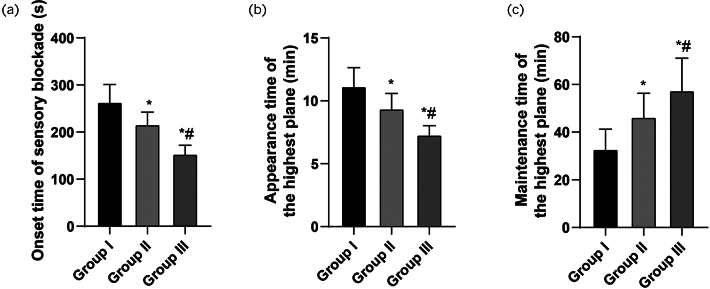
Comparison of sensory blockade effect in the three groups. (a) Onset time of sensory blockade (s). (b) Appearance time of the highest plane (min). (c) Maintenance time of the highest plane (min). *p<0.05 vs Group I; ^#^*p*<0.05 vs Group II.

The onset time of sensory blockade and the appearance time of the highest plane were significantly earlier and the maintenance time of the highest plane was significantly longer in Group II and Group III than in Group I, and the effect was more pronounced in Group III (*p*<0.05; Figure 1).

### Motor blockade effect

**Figure 2:**
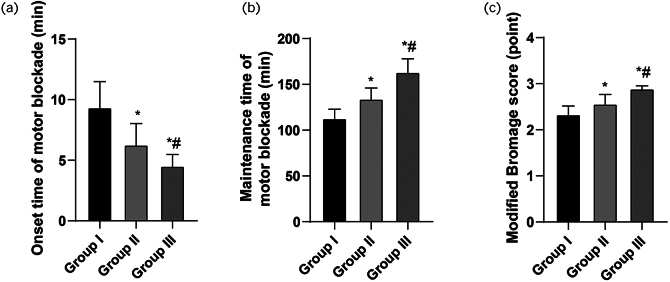
Comparison of motor blockade effect in the three groups. (a) Onset time of motor blockade (min). (b) Maintenance time of motor blockade (min). (c) Modified Bromage score (point). *p<0.05 vs Group I; ^#^*p*<0.05 vs Group II.

The onset time of motor blockade was notably earlier, the maintenance time of the highest plane was markedly longer and the modified Bromage score was distinctly higher in Group II and Group III in comparison with Group I, and the tendency was more marked in Group III (*p*<0.05; Figure 2).

### Haemodynamics

**Figure 3:**
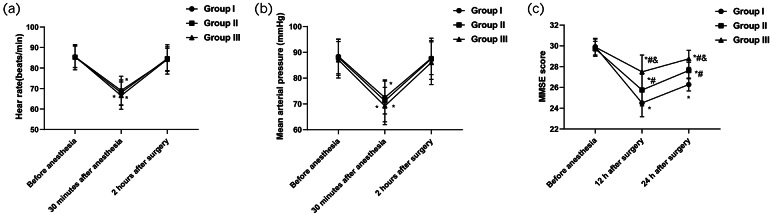
Comparison of haemodynamics and MMSE scores in the three groups. (a) Heart rate (beats/min); (b) Mean arterial pressure (mmHg); (c) MMSE scores. * *p*<0.05 vs before anesthesia; #*p*<0.05 vs Group I; ^&^*p*<0.05 vs Group II. MMSE = Mini Mental State Examination

Before anaesthesia, there was no statistically significant difference in heart rate and MAP among the three groups (all *p*>0.05). Heart rate and MAP of the three groups were reduced 30min after anaesthesia, and returned to normal 2h after surgery. Heart rate and MAP were lower in Group III than in Group I and Group II at 30min after anaesthesia, but there was no statistically significant difference among the three groups (all *p*>0.05) (Figure 3a and b).

### MMSE scores and the incidence of cognitive impairment

Before anaesthesia, the MMSE scores of the three groups were compared, and exhibited no statistical significance (*p*>0.05). The 12- and 24-h postoperative MMSE scores of the three groups were lower than those before anaesthesia. The postoperative 12- and 24-h MMSE scores were higher and the incidence of postoperative 12h-cognitive impairment was lower in Group III than in Group I and Group II. The postoperative 12- and 24-h MMSE scores were higher and the incidence of postoperative 12-h cognitive impairment was lower in Group II in comparison with those in Group I (all *p*<0.05) (Figure 3c; Table 2).

**Table 2:** Comparison of the incidence of cognitive impairment in Groups I–III

	Group I (*n*=51)	Group II (*n*=51)	Group III (*n*=51)
Incidence of cognitive impairment (case)			
12h after surgery	23	11*	3*^,#^
24h after surgery	5	4	0

*p<0.05 vs Group I; ^#^*p*<0.05 vs Group II.

### Adverse reactions

No statistically significant difference in the incidence of adverse reactions in patients among the three groups was found (*p*>0.05) (Table 3).

**Table 3:** Comparison of adverse reactions in Groups I–III

Adverse reactions	Group I (*n* = 51)	Group II (*n* = 51)	Group III (*n* = 51)
Nausea and vomiting	2	3	4
Bradycardia	0	1	1
Hypotension	1	1	3
Respiratory depression	1	1	1
Total adverse effects	4 (7.84%)	6 (11.76%)	9 (19.65%)

## Discussion

Surgery is a treatment used frequently for elderly fractures and can markedly improve the patient’s activities of daily living, which is of great clinical significance. Anaesthesia is a prerequisite for surgery, and the effects of anaesthesia are different for different kinds or doses of anaesthetic drugs and different anaesthesia methods. In our paper, we aimed to unravel the effectiveness of epidural anaesthesia induced by different dosages of ropivacaine combined with lidocaine in geriatric orthopaedic surgery. In detail, elderly orthopaedic surgical patients who underwent epidural anaesthesia were allocated into three groups following the use of different dosages of ropivacaine: Group I (0.375% ropivacaine+1% lidocaine), Group II (0.50% ropivacaine+1% lidocaine) and Group III (0.75% ropivacaine+1% lidocaine) to unveil differences in sensory and motor blockade effects, haemodynamics, cognitive function and occurrence of cognitive impairment, and adverse reactions.

Clinically, short-acting anaesthetics combined with long-acting anaesthetics can be utilised to improve, accelerate or prolong neural blockade.^18^ As previously reported, lidocaine combined with bupivacaine has long been applied for spinal anaesthesia, which reduces the spinal block duration, along with a shorter recovery time.^19,20^ Another article has demonstrated that combined spinal anaesthesia with ropivacaine and lidocaine has been utilised in interscalene blocks.^21^ Nevertheless, it remains unclear whether lidocaine combined with ropivacaine enhances the treatment effect in geriatric orthopaedic surgery. In our study, we found that epidural anaesthesia combining 0.75% ropivacaine with 1% lidocaine has a rapid onset of action, a long duration of anaesthesia maintenance, a low overall impact on cognitive function and good medication safety. Ropivacaine may be effective as a local anaesthetic for digital nerve blocks, which could be utilised for prolonged surgery without additional injections and offers lasting postoperative analgesia.^12^ Epidural 0.2% ropivacaine has been disclosed to be effective in the starting and maintenance of labour analgesia and for pain relief after abdominal or orthopaedic surgery, particularly when administrated in combination with opioids.^22^ Intravenous lidocaine has recently been utilised broadly as a propofol-adjuvant drug in anaesthesia to diminish pain, reduce the consumption of opioids or sedatives and accelerate the recovery of bowel function after surgery.^23^ There is evidence that intravenous lidocaine decreases postoperative pain intensity together with opioid consumption and shortens hospital stays in elderly patients.^24^ Furthermore, lidocaine serves as an effective neuroprotective agent for the therapy of early cognitive dysfunction after surgery in elderly patients receiving spine surgery.^25^ Similar to our findings, Te *et al* have found that patients treated with ropivacaine plus lidocaine exhibit reduced postoperative pain scores at all timepoints, with longer mean time to the first postoperative analgesia scores.^16^

Our study highlighted that the onset time of sensory blockade and the appearance time of the highest plane were significantly earlier and the maintenance time of the highest plane was significantly longer in Group II and Group III than in Group I; the onset time of motor blockade was notably earlier, the maintenance time of the highest plane was markedly longer, and the modified Bromage score was distinctly higher in Group II and Group III in comparison with Group I, and the tendency was more obvious in Group III. Additionally, the differences in heart rate and MAP before anaesthesia, 30min after anaesthesia and 2h after surgery were not statistically significant among the three groups. The postoperative 12- and 24-h MMSE scores were higher and the incidence of postoperative 12h-cognitive impairment was lower in Group III than in Group I and Group II. Moreover, no statistically significant differences in the incidence of adverse reactions in patients among the three groups were found. All these findings validate the best effect of 0.75% ropivacaine. In line with our findings, previous research has suggested that, in lower impacted third molar surgery, 0.75% ropivacaine may effectively supply prolonged postoperative analgesia, adequate anaesthesia and better postoperative pain control, as well as a safer cardiovascular profile.^26^ Jaichandran *et al* have concluded that 0.75% ropivacaine is a better local anaesthetic solution of choice for patients receiving primary vitreoretinal surgery in comparison with 0.5% bupivacaine.^27^ A level 1 study has unveiled that after knee arthroscopic surgery, 0.75% ropivacaine exhibits better intraarticular analgesia than 0.5% of bupivacaine.^28^

To conclude, this paper highlights that epidural anaesthesia combining 0.75% ropivacaine combined with 1% lidocaine in geriatric orthopaedic surgery performs a rapid onset of action, a long duration of anaesthesia maintenance, a low overall impact on cognitive function and good medication safety. Clinically, appropriate anaesthesia methods should be selected according to the specific situation of patients to reduce the occurrence of anaesthesia-related complications. Meanwhile, although the sample size of this study is sufficient, it is still relatively small. In future work, we will include a larger sample size for related studies.
